# Expression of Wnt and Notch signaling pathways in inflammatory bowel disease treated with mesenchymal stem cell transplantation: evaluation in a rat model

**DOI:** 10.1186/s13287-015-0092-3

**Published:** 2015-05-22

**Authors:** Yanfen Xing, Xiaojie Chen, Yanwen Cao, Jianyun Huang, Xuhong Xie, Yaming Wei

**Affiliations:** Department of Blood Transfusion, Guangzhou First People’s Hospital, Guangzhou Medical University, No. 1. Panfu Road, Guangzhou, 510180 Guangdong Province China; Guangdong Key laboratory of Clinical Molecular Medicine and Diagnostics, Guangzhou First People’s Hospital, Guangzhou Medical University, No. 1. Panfu Road, Guangzhou, 510180 Guangdong Province China; Department of Gastroenterology, Guangzhou First People’s Hospital, Guangzhou Medical University, No. 1. Panfu Road, Guangzhou, 510180 Guangdong Province China

## Abstract

**Introduction:**

The purpose of this study was to investigate the expression of Wnt and Notch signaling pathway-related genes in inflammatory bowel disease (IBD) treated with mesenchymal stem cell transplantation (MSCT).

**Methods:**

TNBS (2,4,6-trinitrobenzene sulfonic acid) was used to establish IBD in a rat model. Mesenchymal stem cells (MSCs) were transplanted via tail vein transfusion. Saline water was used in a control group. The expression of Wnt and Notch main signaling molecules was screened by gene chips and verified by quantitative reverse transcription-polymerase chain reaction in the IBD rat model on day 14 and day 28 after transplantation.

**Results:**

The IBD rat models were successfully established and MSCs were transplanted into those models. Genome-wide expression profile chips identified a total of 388 differentially expressive genes, of which 191 were upregulated and 197 were downregulated in the MSC-transplanted group in comparison with the IBD control group. Real-time quantitative polymerase chain reaction results showed that the level of *Olfm4* mRNA expression in the IBD group (2.54±0.20) was significantly increased compared with the MSCT group (1.39±0.54) and the normal group (1.62±0.25) (*P* <0.05). The *Wnt3a* mRNA was more highly expressed in IBD rats (2.92±0.94) and decreased in MSCT rats (0.17±0.63, *P* <0.05). The expression of *GSK-3β* mRNA was decreased in the setting of inflammation (0.65±0.04 versus 1.00±0.01 in normal group, *P* <0.05) but returned to normal levels after MSCT (0.81±0.17). The expression of *β-catenin* was observed to increase in IBD tissues (1.76±0.44) compared with normal tissues (1.00±0.01, *P* <0.05), but no difference was found in the MSCT group (1.12±0.36). *Wnt11* declined at 14 days and returned to normal levels at 28 days in the IBD group; in comparison, a significantly lower expression was found in MSCT rats. There were no differences in the expression of *Fzd3*, *c-myc*, *TCF4*, and *Wnt5a* in inflammation, but all of those genes declined after MSCT treatment.

**Conclusions:**

The canonical Wnt and Notch signaling pathways are activated in IBD and may be suppressed by stem cell transplantation to differentiate into intestinal epithelium after MSCT. Moreover, the non-canonical Wnt signaling may be inhibited by canonical Wnt signaling in the setting of inflammation and may also be suppressed by MSCT.

## Introduction

Inflammatory bowel disease (IBD) includes ulcerative colitis (UC) and Crohn’s disease (CD). These conditions produce recurrent chronic inflammatory illnesses of the intestinal tract. Studies show that IBD comprises heterogeneous disorders of various etiologies, in which hereditary predisposition, environmental factors, and abnormal immune response work together to induce this disease [1, 2]. Currently, the conventional medications are salicylic acid, corticosteroids, immunosuppressive agents, and antibiotics [3]. Although these therapies may offer temporary remission, curative effect is not very obvious, and adverse reactions [4] such as psoriasis [5], drug-induced cytotoxicity [6], and hypersensitivity [7] may arise in response to treatment.

Mesenchymal stem cells (MSCs) have proliferation, differentiation, and engraftment capacity in appropriate target tissues [8]. Several studies have reported the MSCT in the treatment of UC and CD could restore epithelial barrier integrity[8], induce immune suppression [9, 10], and stimulate regeneration of endogenous host progenitor cells [11]. In a previous study, we confirmed by *in situ* hybridazation and immunohistochemistry that allogenic transplanted hematopoietic stem cells or MSCs could populate the injured regions of the colon [12]. MSCs not only stimulated progenitor cells to improve epithelial renewal but also engrafted in the damage tissues and even differentiated into colonic interstitial cells [13]. However, the mechanisms are not clear. In particular, we do not know which genes and pathways are involved in reparation of the mucosa and transformation into intestinal epithelial cells.

The Wnt signaling pathway and Notch signaling pathway are major pathways in stem cell proliferation and differentiation capacity. Wnt signaling is classified as two types: canonical pathway and non-canonical pathway [14]. Wingless-type mouse mammary tumor virus (MMTV) integration site family, member 3A (*Wnt3a*) activates the canonical Wnt signaling pathway, which is a prominent member of the Wnt family. The other members of the Wnt ligand family—such as Wingless-type MMTV integration site family, member 5A (*Wnt5a*) and Wingless-type MMTV integration site family, member 11 (*Wnt11*)—activate the non-canonical Wnt signaling pathway [15]. The Notch signaling pathway can be activated by inhibiting atonal homolog 1 (*Atoh1*) gene expression, which determines cell fate [16]. The Wnt signaling pathway can promote progenitor cell proliferation, maintain a cycling cell type, and prevent differentiation. Meanwhile, the Notch pathway has the capacity to maintain the progenitor cells in an undifferentiated, proliferating state [17]. Wnt and Notch pathways are very important in the regulation of cell fate. These pathways have been widely investigated in normal tissues but not in inflammatory colonic tissues or in the setting of colonic tissue regeneration secondary to MSCT.

In this study, we explored the differential expression of genes associated with the Wnt and Notch signaling pathways in MSCT of an IBD rat model. Using a cDNA chip, we verified these related genes by quantitative reverse transcription-polymerase chain reaction (RT-PCR). We will discuss the mechanism of differentiation of implanted MSCs in the repair of damaged intestinal epithelium.

## Methods

### Animals

Eighteen Sprague Dawley male rats weighing 180 to 200 g were maintained in accordance with the guidelines of the Committee for Animal Research of Guangzhou Medical University. This study was conducted in accordance with the Regulations for the Administration of Affairs Concerning Experimental Animals. All animal experiments were approved by the Guangdong Medical Laboratory Animal Center. Eighteen male rats were randomly divided into three groups, and each group included six rats. There are three groups: normal control group, IBD group, and MSC transplantation group (MSCT group).

### Cells

MSCs, which were labeled with green fluorescent protein (GFP), were obtained from Cyagen Biosciences Inc. (Santa Clara, CA, USA). MSCs were re-suspended at 37 °C, 5 % CO_2_ in low-glucose Dulbecco’s modified Eagle’s medium (Gibco, New York, NY, USA) containing 10 % fetal bovine serum (Gibco) and 100 μg/ml penicillin and streptomycin (Solarbio, Beijing, China). The medium was changed every 1 to 3 days. After two or three passages, MSCs were used for transplantation to treat IBD. The immunophenotyping of MSCs was analyzed by flow cytometry for CD44^+^ and CD54^+^ antigen markers. When MSCs covered 80 % to 90 % of the area of the culture bottle, they were passaged. Transplantation was performed with passage 3 to passage 5 cells.

### Experimental inflammatory bowel disease models

IBD experimental colitis rats model were induced by delivering 1.0 ml of 20 mg 2,4,6-trinitrobenzene sulfonic acid (TNBS) (Sigma-Aldrich, St. Louis, MO, USA) in 50 % ethanol solution. A rubber infusion tube was positioned 8 cm from the anus to instill TNBS into the lumen of the colon. To ensure allocation of TNBS within the entire colon, rats were maintained vertically for 1 minute. The normal group received 0.1 ml of normal saline.

### Green fluorescent protein-labeled mesenchymal stem cell transplantation models

MSCs were transfected by virus to stable GFP fluorescence expression. Before MSCT models were established, the concentration of MSCs was adjusted to 2×10^6^/ml. After 24-hour IBD model yielding, the rat tail was wiped with a warm towel and alcohol for vein dilation. Then 1.0 ml of previous MSCs was transplanted via the tail vein. Removal of colon tissue lesions from normal rats, IBD rats, and MSCT rats was done at 14 and 28 days after MSCT. Samples, which covered lesion region, were removed from colon tissue 8 cm far from anus.

### Histopathology

Four to six rats were killed on days 14 and 28 in the IBD and MSCT groups. First, the colon tissues were observed with the naked eye and with a microscope. Second, the lesion tissues were fixed in 4 % buffered formaldehyde and embedded in paraffin wax; histological samples were cut from paraffin-embedded blocks and stained with hematoxylin and eosin for light microscope and fluorescent microscope examination.

### Genome-wide expression profile chip analysis

Total RNA was extracted from IBD colorectal tissues and colorectal tissues at 28 days after MSCT and transcribed into cDNA. The cDNA was scanned by the Axon GenePix 400B microarray analysis scanner (Axon Instruments, part of Molecular Devices, Sunnyvale, CA, USA). Data were analyzed by NimbleScan version 2.6 software (NimbleGen, part of Roche, Basel, Switzerland) (screening parameters are *P* <0.05 and fold change (FC) >2). Mircoarray data are available at Gene Expression Omnibus under accession number GSE68653.

### RNA isolation and real-time polymerase chain reaction

Total RNA was extracted from MSCs, normal colorectal tissues, IBD colorectal tissues, and colorectal tissues at 28 days after MSCT by using RNAiso Plus reagent in accordance with the instructions of the manufacturer (Takara, Otsu, Japan). The PrimeScript RT Master Mix (Perfect Real Time) (Clontech, part of Takara) and 500 ng of total RNA and poly-dT primers were used for synthesis of cDNA. All gene-specific primers listed in Table 1 were deposited in the Takara Corporation. Quantitative RT-PCR was carried out by using SYBR Premix Ex Taq™ (Takara) and performed by using two-step amplification. PCR amplification conditions were as follows: 95 °C for 30 seconds (one cycle), 95 °C for 3 seconds, and 60 °C for 30 seconds (40 cycles). The crossing threshold values were obtained during the exponential amplification phase by using the ABI 7500 Fast Real-Time PCR system (Applied Biosystems, a brand of Thermo Fisher Scientific, Waltham, MA, USA).

**Table 1:** Primers used in quantitative reverse transcription-polymerase chain reaction

Gene	Forward primer (5′-3′)	Reverse primer (5′-3′)
*Wnt3a*	CATCGCCAGTCACATGCACCT	CGTCTATGCCATGCGAGCTCA
*Wnt5a*	GCCACTTGTATCAGGACCACA	GGCATTTACCACTCCAGCAG
*Wnt11*	TACCTGCTTGACCTGGAGAG	TAGGAGCATCGGAAAACTTG
*Fzd3*	GTGTGTTTTGTCGGCCTCTACG	GAATGTGATACTCTCTGCAGCGTTC
*β-catenin*	CTGACCAAACTGCTAAATGACG	GATGGTGGGAAAGGTTGTGTAG
*GSK-3β*	TCGCCACTCGAGTAGAAGAAA	ACTTTGTGACTCAGGAGAACT
*C-myc*	TCTTGGAACGTCAGAGGAGAA	GCTTGAACGGACAGGATGTAG
*Tcf4*	GCCTCTCATCACGTACAGCA	GGATGGGGGATTTGTCCTAC
*APC*	TGGAATGGTGAGTGGGATTG	GGGTCTTGCTTCTGCTTGGT
*Cyclin D*	AGGCGGATGAGAACAAGCA	GAGGGTGGGTTGGAAATGAA
*Atoh1*	CCAGGGTGAGCTGGTAAGG	CGTTGTTGAAGGACGGGAT
*Olfm4*	GCCAGCACTGGTAACATA	GCGAGTAGCGAAAGAAC
*β-actin*	GTAAAGACCTCTATGCCAACA	GGACTCATCGTACTCCTGCT

### Statistical analysis

Experiments were reproduced three times. In Table 1, quantifiable determinations are expressed as mean ± standard error of the mean of the indicated number of experiments. Significance of differences was evaluated by one-way analysis of variance, followed by the least significant difference (LSD) test and Dunnett T3 test when evaluating more than two conditions. Significant changes are indicated as follows: *P <0.05, **P <0.01.

## Results

### Macroscopic evaluation of TNBS-induced colitis in rat

**Fig. 1:**
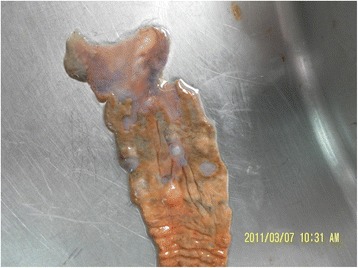
Photograph of 2,4,6-trinitrobenzene sulfonic acid (TNBS)-induced colitis at 14 days in a rat model

Anatomic examination revealed pronounced colonic damage, including bowel wall edema, luminal dilation, mucosal erosions and ulcers, and even perforation (Fig. 1). We also checked normal tissue from the same anima and no obviously inflammation was found in that region (data not shown).

### Histopathologic analysis of colonic epithelium in inflammatory bowel disease rat model

**Fig. 2:**
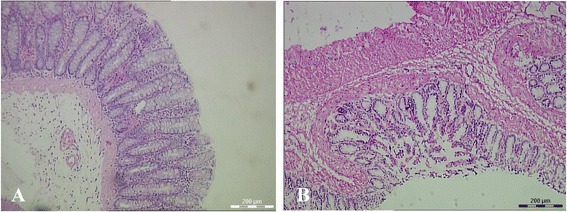
Characterization of 2,4,6-trinitrobenzene sulfonic acid (TNBS)-induced colonic epithelium damage and normal tissues at 14 days. **a** Normal tissues under the microscope (hematoxylin and eosin (H&E), ×100). **b** Induced inflammatory bowel disease in rats (H&E, ×100)

Histochemical analysis of the colonic tissues revealed mucosal lesions, edema, ulcers, and intense inflammatory cell infiltrate in TNBS-induced colonic epithelium (Fig. 2). The histological score was similar to our previously study and is not shown here [12]. In the lamina propria, crypt and mucosa caused serious inflammation, including diffusion of granulocytes, leukocytes, and histiocytes; gland damage; and goblet cell reduction or even disappearance. More detailed results were described in our previous study [12].

### Mesenchymal stem cells engraft in lesion tissues and repair intestinal epithelium in an inflammatory bowel disease rat model

**Fig. 3:**
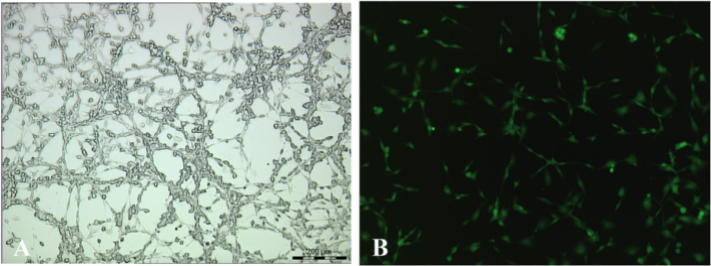
The morphology of mesenchymal stem cells (MSCs) observed under microscope. **a** Well-grown MSCs under microscope (×100). **b** Stable expression of green fluorescent protein was observed in MSCs under fluorescent microscope (×100)

**Fig. 4:**
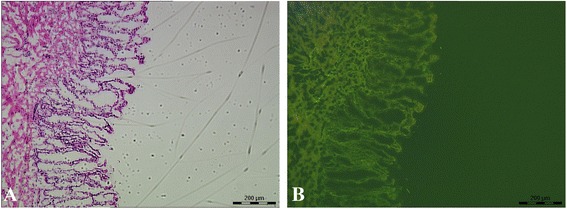
Observation of colon tissue at 28 days after mesenchymal stem cell transplantation (MSCT) under microscope and fluorescent microscope. **a** Colon tissue lesion significantly improved after MSCT (hematoxylin and eosin, ×100). **b** The fluorescence signal could be observed in each layer of intestinal tissue (×100)

The result of flow cytometry showed that MSCs expressed CD44^+^ and CD54^+^ antigen markers. The positive cells of CD44^+^ were 89.6±6.0 %, and those of CD54^+^ were 95.5±3.9 % [12]. Well-grown MSCs with stable expression of GFP were cultured (Fig. 3) and then transplanted into established IBD rats. The pathological manifestations of IBD in the MSCT group were better than those in the IBD group; most ulcers were healed, but edema and inflammatory cell infiltration still existed (Fig. 4a). Under fluorescent microscope, MSCs labeled by GFP were populated in the damaged areas from the basement to the top of the fossae. GFP-positive cell density accounted for more than 50 % of the epithelial cells of the colorectal mucosa (Fig. 4b). In our previous study, the *Sry* gene and Y chromosome were detected by PCR and fluorescence *in situ* hybridization (FISH) to determine the location of male donor cells in the female colon after transplantation [12].

### Genome-wide expression profile chips

At 14-day treatment with MSCT, main lesion colon tissues were collected for chip analysis. Genome-wide expression profile chips showed very good sample data trend and study repeatability. When *P* <0.05 and FC >2 were screened for, a total of 388 differentially expressive genes were found; of these, 191 were upregulated and 197 downregulated in the MSCT group in comparison with the IBD control group. These genes were involved mainly in inflammatory reaction, immune reaction, and cell differentiation. The top 10 and bottom 10 genes are shown in Tables 2 and 3, respectively.

**Table 2:** Top 10 expression genes analyzed by genome-wide expression profile chips in inflammatory bowel disease rats at 14 days after treatment with mesenchymal stem cell transplantation

Gene ID	Official symbol	Official full name	Fold changes
29266	*Mcpt2*	Mast cell protease 2	+21.28
29265	*Mcpt1*	Mast cell protease 1	+18.99
360922	*Igj*	Immunoglobulin joining chain	+17.38
56266	*Cyp4f1*	Cytochrome P450, family4, subfamily f, polypeptide 1	+17.98
24360	*Fabp1*	Fatty acid binding protein 1	+16.09
689116	*Pigz*	Phosphatidylinositol glycan anchor biosynthesis, class z	+15.87
290409	*Olfm4*	Olfactomedin 4	+14.46
286976	*Gcnt3*	Glucosaminyl (N-acetyl) transferase 3	+11.87
299354	*Ighg*	Immunoglobulin heavy chain	+11.34
36672	*LOC366772*	Similar immunoglobulin heavy chain	+10.30

**Table 3:** Bottom 10 expression genes analyzed by genome-wide expression profile chips in inflammatory bowel disease rats at 14 days after treatment with mesenchymal stem cell transplantation

Gene ID	Official symbol	Official full name	Fold changes
679314	*LOC679314*	Hypothetical protein LOC679314	−22.54
24249	*S100g*	S100 calcium binding protein G	−8.58
298873	*Slc7a15*	Solute carrier family 7, member 15	−6.99
81828	*Zp2*	Zona pellucida glycoprotein 2	−5.99
287644	*Phospho1*	Phosphatase, orphan 1	−5.88
155205	*Slc36a1*	Solute carrier family 36, member 1	−5.51
500973	*Slc37a2*	Solute carrier family 37, member 2	−4.62
361296	*Rnf125*	Ring finger protein 125	−4.46
308134	*Tmem35*	Transmembrane protein 35	−4.31
113902	*Ces3*	Carboxylesterase3	−4.15

In all 20 genes, atonal homolog 1 (*Atoh1*) and olfactomedin 4 (*Olfm4*) were upregulated and associated with the Notch signaling pathway. Wnt11 was downregulated and related to the non-canonical Wnt signaling pathway. Other genes involved in the Wnt/β-catenin signaling pathway, such as *Wnt3a*, *Wnt5a*, frizzled family receptor 3 (*Fzd3*), glycogen synthase kinase-3β (*GSK-3β*), β-catenin, myelocytomatosis oncogene (*C-myc*), cyclin D, T-cell factor 4 (*Tcf4*), and adenomatous polyposis coli (*APC*), were also detected

### Notch signaling pathway in mesenchymal stem cell transplantation repairing inflammatory bowel tissue

**Fig. 5:**
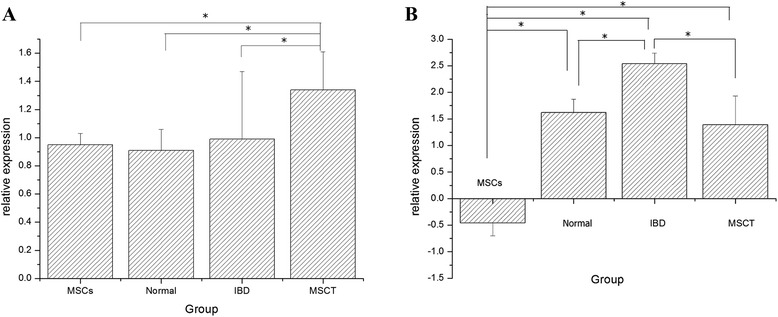
Expression of atonal homolog 1 (*Atoh1*) and olfactomedin 4 (*Olfm4*) genes in inflammatory bowel disease (IBD) rats at 14 days after treatment with mesenchymal stem cell transplantation (MSCT). **a** Expression of *Atoh1* in MSCT group upregulated in comparison with the other groups. **b** Expression of *Olfm4* increased in IBD group. MSC, mesenchymal stem cell. *P<0.05

*Atoh1* and olfactomedin 4 (*Olfm4*) were associated with the Notch signaling pathway, which regulated cell proliferation and differentiation [18, 19]. The expression of *Atoh1* in the MSCT group was significantly higher than in the MSC (1.34±0.27 versus 0.95±0.08, *P* = 0.011), normal (1.34±0.27 versus 0.91±0.15, *P* = 0.004), or IBD rat models (1.34±0.27 versus 0.99±0.48, *P* = 0.007) (there were no significant differences among these three groups). The expression of *Olfm4* in MSCT rats was downregulated in comparison with IBD rats (*P* <0.05). No difference was found when compared with normal rats (*P* >0.05) (Table 4, Fig. 5).

**Table 4:** Expression of *Atoh1* and *Olfm4* genes (χ±s)

Group	Number	*Atoh1*	*Olfm4*
MSCs	3	0.95±0.08	−0.46±0.24^b,c^
Normal	3	0.91±0.15	1.62±0.25^a,c^
IBD	9	0.99±0.48	2.54±0.20^a,b^
MSCT	9	1.34±0.27^a-c^	1.39±0.54^a,c^

^a^ *P* <0.05 versus mesenchymal stem cell (MSC) group; ^b^ *P* <0.05 versus normal group; ^c^ *P* <0.05 versus inflammatory bowel disease (IBD) group. MSCT, mesenchymal stem cell transplantation

### Canonical Wnt/β-catenin signaling pathway in inflammatory bowel disease and mesenchymal stem cell transplantation groups

**Fig. 6:**
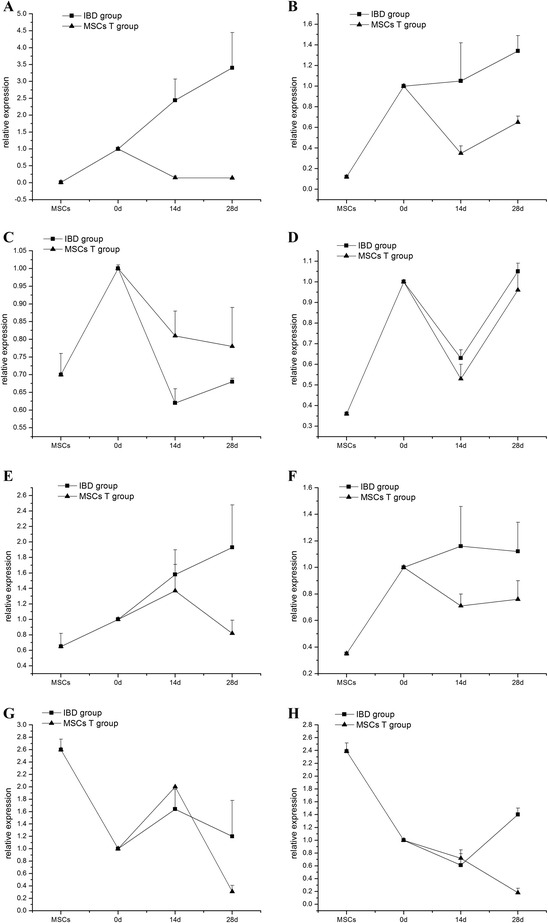
Expression of canonical Wnt signaling-related genes in mesenchymal stem cell transplantation (MSCT) group compared with inflammatory bowel disease (IBD) rat model. **a** *Wnt3a* mRNA decreased expression in MSCT rats compared with IBD rats (*P* = 0.004). **b** Declined *Fzd3* expression (*P* = 0.014). **c** Decreased *GSK-3β* mRNA expression in inflammation (*P* = 0.035) and recovery to normal level after MSCT. **d** Decreased expression of *APC* at 14 days (*P* = 0.034), increased to normal level at 28 days. **e** Lower *β-catenin* mRNA expression in MSCT rats (*P* <0.001). **f** No different expression of *TCF4* between IBD group and normal group, and low expression in mesenchymal stem cells (MSCs) (*P* = 0.036). **g** High *c-myc* expression at 14 days (*P* <0.01) declined at 28 days (P = 0.024). **h** Reduced *cyclin D* expression in MSCT at 28 days (*P* = 0.02)

Genes of the canonical pathway were verified by RT-PCR. The expression of *Wnt3a* and *β-catenin* increased significantly, and *GSK-3β* decreased in the IBD rat group, compared with normal rats, at 14 and 28 days. Adenomatous polyposis coli (*APC*) and *cyclin D* exhibited downregulated expression in the IBD rat group at 14 days and demonstrated upregulation at 28 days compared with the normal group. There were no differences in the expression of *Fzd3*, *TCF4*, and *c-myc* between IBD model rats and the normal group (Table 5, Fig. 6).

**Table 5:** Expression of canonical pathway-related genes (χ±s)

Group	Number	*Wnt3a*	*Fzd3*	*GSK-3β*	*APC*	*β-catenin*	*TCF4*	*C-myc*	*Cyclin D*
MSCs	3	0.01±0.01^b,c^	0.12±0.01^b,c^	0.70±0.06^b^	0.36±0.01^b,c^	0.65±0.17^c^	0.35±0.01^b,c^	2.60±0.17^b^	2.39±0.13^b,c^
Normal	6	1.00±0.01^a,c^	1.00±0.01^a^	1.00±0.01^c^	1.00±0.01^a^	1.00±0.01^c^	1.00±0.01^a^	1.00±0.01^a^	1.00±0.01^a^
14-day IBD	3	2.44±0.63^a,b^	1.05±0.37^a^	0.62±0.04^b^	0.63±0.04^a,b^	1.58±0.32^a,b^	1.16±0.30^a^	1.64±0.32	0.61±0.24^a,b^
14-day MSCT	3	0.15±0.05^a-c^	0.35±0.07^a-c^	0.81±0.07^c^	0.53±0.07^b^	1.37±0.34^a^	0.71±0.09^a-c^	2.00±0.01^b^	0.72±0.07^a^
28-day IBD	3	3.40±1.05^a,b^	1.34±0.15^a^	0.68±0.01^b^	1.05±0.01^a^	1.93±0.55^a,b^	1.12±0.22^a^	1.20±0.58	1.40±0.10^a^
28-day MSCT	3	0.14±0.02^a-c^	0.65±0.06^a-c^	0.78±0.11^c^	0.96±0.13	0.82±0.17^c^	0.76±0.14^a-c^	0.31±0.10^a-c^	0.18±0.07^a-c^

^a^ *P* <0.05 versus mesenchymal stem cell (MSC) group; ^b^ *P* <0.05 versus normal group; ^c^ *P* <0.05 versus inflammatory bowel disease (IBD) group. MSCT, mesenchymal stem cell transplantation

The gene expressions in MSCT rats are shown in Fig. 6. The expression of *Wnt3a*, *Fzd3*, *TCF4*, and *cyclin D* significantly declined after MSCT, compared with the IBD group without MSCT, whereas expression of *GSK-3β* was upregulated to normal levels and was higher than in IBD rat models. In the MSCT group, *β-catenin* expression was lower than that of the IBD rat group at 28 days. *APC* expression did not change in the MSCT group. *C-myc* exhibited increased expression at 14 days and decreased expression at 28 days, compared with the IBD group, whereas *c-myc* and *cyclin D* showed high expression, and other genes demonstrated comparatively lower expression in MSCs.

### Non-canonical Wnt signaling pathway in inflammatory bowel disease and mesenchymal stem cell transplantation groups

**Fig. 7:**
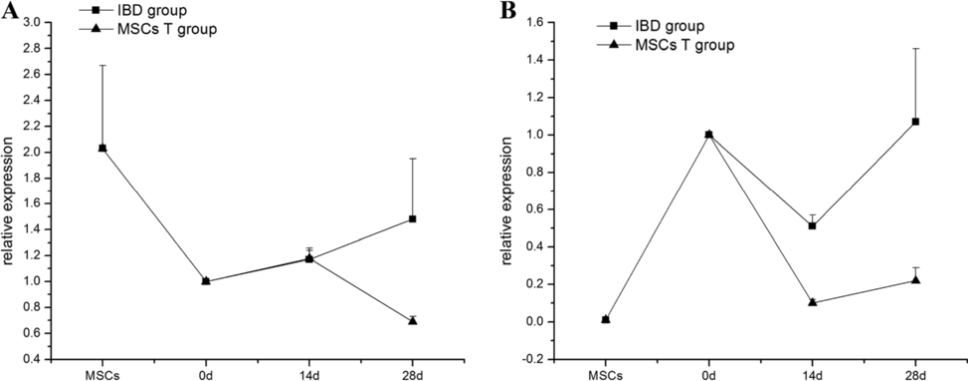
Expression of non-canonical Wnt signaling genes in mesenchymal stem cell transplantation (MSCT) group compared with inflammatory bowel disease (IBD) rat model. **a** Declined *Wnt5a* mRNA expression on day 28 in the MSCT group. **b** Lower expression of *Wnt11* in MSCT rats. MSC, mesenchymal stem cell

*Wnt5a* and *Wnt11*, two main genes in the non-canonical Wnt signaling pathway, were screened with RT-PCR. *Wnt5a* did not change significantly at 14 days and showed lower expression at 28 days in the MSCT group (*P* = 0.04). *Wnt11* showed lower expression in the MSCT group compared with the IBD group at 14 and 28 days (*P* = 0.048); in the IBD model, *Wnt11* decreased at 14 days (*P* = 0.019) and was restored to normal level at 28 days (*P* >0.05) (Table 6, Fig. 7).

**Table 6:** Expression of *Wnt5a* and *Wnt11* genes (χ±s)

Group	Number	*Wnt5a*	*Wnt11*
MSCs	3	2.03±0.64	0.01±0.003^b^
Normal	6	1.00±0.01	1.00±0.01^a,c^
14-day IBD	3	1.17±0.09	0.51±0.06^a,b^
14-day MSCT	3	1.18±0.06	0.10±0.02^b^
28-day IBD	3	1.48±0.47	1.07±0.39^a,c^
28-day MSCT	3	0.69±0.04^a-c^	0.22±0.07^b^

^a^ *P* <0.05 versus mesenchymal stem cell (MSC) group; ^b^ *P* <0.05 versus normal group; ^c^ *P* <0.05 versus inflammatory bowel disease (IBD) rat model. MSCT, mesenchymal stem cell transplantation

## Discussion

The intestinal stem cells showed renewal, proliferation, and differentiation capacity and were able to restore the intestinal mucosa [11]. Transplanted MSCs demonstrated the ability to engraft into the intestinal mucosa [12, 20] and alleviate the autoimmune response, facilitating the treatment of IBD [21]. Previous studies demonstrated that MSCs could engraft into the damaged area of intestinal epithelium tissues, showing characteristics of epithelial cells [22, 23].

Intestinal stem cells have several signaling pathways, such as Wnt, BMP, PI3K/Akt, Hedgehog, and Notch pathways. Wnt and Notch signaling pathways play important roles in determining intestinal stem cell fate, including maintenance of stem cell homeostasis, inhibition of cell differentiation, and control of Paneth cell development [24]. Meanwhile, those pathways are also crucial in MSCs, which regulate self-renewal and differentiation [25]. Canonical Wnt signaling activated by *Wnt3a* enhances the proliferation of MSCs and suppresses MSC osteogenic differentiation, whereas non-canonical Wnt signaling activated by *Wnt5a* maintains MSC numbers and promotes osteogenesis [26]. The Notch pathway also regulates MSC fate and osteoblastic differentiation [27]. In recent years, *Wnt3a* and *Fzd3* were reported to increase expression in IBD colon tissues compared with non-IBD colon tissues [28]. The protein level of *β-catenin* was found to increase in colonic epithelial crypts from damaged mucosa [29].

In our study, we successfully established the IBD and MSCT model rats and then found that MSCs labeled with green fluorescence were populated in the damaged areas after MSCT. In our previous study, we have demonstrated that hematopoietic stem cells or MSCs could be implanted in the ulcerated experimental colitis region of a rat model after transplantation; male donor cells were followed by *Sry* gene using PCR and Y chromosome FISH in female recipients, and the donor cell rate was evaluated by brown positive cells labeled by bromodeoxyuridine (BrdU) immunohistochemistry. MSCs not only were engrafted in the damaged areas but also were the major source for intestinal epithelia renewal [12]. The other research also proved that MSCs transplanted into gastric tissue, and the beneficial effects might be mediated by MSCs differentiated into gastric interstitial cells [30]. In this study, the fluorescence signal cell was observed in all views of each layer of intestinal tissue, and the vast majority of cells in the intestinal lesion tissue were derived from GFP-labeled MSCs. When intestinal epithelium was inflamed, we found that canonical Wnt signaling was activated by *Wnt3a* and inhibited by *GSK-3β* and *APC*, and the transcription of downstream-related factors would be regulated. These changes promoted intestinal stem cell proliferation and repaired the intestinal mucosa. On the other hand, the expression of *Fzd3*, *c-myc*, and *cyclin D* did not change significantly in IBD rats. These results suggest that *Wnt3a* and *Fzd3* may not work together in activating canonical Wnt signaling to accelerate intestinal stem cell proliferation by promoting the transcription of *c-myc* and *cyclin D*. After MSCT, canonical Wnt signaling-related genes such as *Wnt3a*, *Fzd3*, *β-catenin*, *TCF4*, and *cyclin D* exhibited downregulation, and *GSK-3β* exhibited upregulation, compared with IBD rats; the canonical Wnt signaling pathway may be inhibited when treated with MSCs.

The expression of *APC* differed from that of *c-myc. APC* declined on day 14 and increased to normal levels on day 28 in IBD rats after MSCT. The expression of *c-myc* was increased on day 14 and decreased on day 28 in the MSCT group. *C-myc* was reportedly the target gene of the *APC* pathway [31] and showed high expression in MSCs. Presumably, MSCT might accelerate the expression of *c-myc* in the early stage, inhibiting *APC* expression, and promoting the proliferation of the MSC itself. On day 28, the decreased expression of *c-myc* promoted the differentiation of MSCs into intestinal stem cells.

In the non-canonical Wnt signaling pathway, *Wnt11* can either activate or inhibit the canonical pathway [32, 33]. In our research, expression of *Wnt11* decreased on day 14 and increased to normal levels on day 28. But the expression of *Wnt5a* did not differ between the IBD group and the normal group. In IBD rats treated with MSCT, both *Wnt5a* and *Wnt11* expressions were lower than in IBD and normal rats. We speculated that the non-canonical Wnt signaling activated by *Wnt11* may have been inhibited by canonical signaling in IBD, but the non-canonical Wnt signaling activated by *Wnt5a* did not change in inflammatory conditions. Regardless of which signaling molecule activated the non-canonical signaling pathway, both may be inhibited after MSCT.

*Olfm4* was the target gene of Notch signaling and participated in stem cell fate. The expression of *Olfm4* in the intestine rapidly decreased after Notch inhibition, and increased *Olfm4* expression was observed after activation of Notch signaling [34]. In the present study, the expression of *Olfm4* significantly increased in IBD rats compared with normal rats and MSCT rats. This increased expression in IBD is consistent with the conclusions of Gersemann *et al*. [35]. *Atoh1* was an inhibitor of the Notch signaling pathway and essential to the differentiation of intestinal stem cells [36]. After MSCT, expression of *Olfm4* was downregulated and *Atoh1* was upregulated. This suggests that Notch signaling may be suppressed in the process of MSCT treatment.

## Conclusions

In this study, we investigated the effect of Wnt and Notch signaling in the process of MSCT repair of inflammatory bowel disease in a rat model. Our results suggest that canonical Wnt and Notch signaling pathways are activated to promote the proliferation of intestinal stem cells in IBD, countering inflammation. Non-canonical Wnt signaling may be suppressed by canonical signaling during inflammation. In treatment with MSCs, Notch and Wnt signaling pathways are inhibited to prevent stem cell proliferation and promote MSC differentiation to intestinal epithelial cells. Shortly after MSCT, the high expression of *c-myc* and low expression of *APC* mRNA facilitate MSC proliferation, and then differentiation into intestinal epithelial cells in the anaphase, by reducing the expression of *c-myc*.

## Abbreviations

APC: Adenomatous polyposis coli
Atoh1: Atonal homolog 1
CD: Crohn’s disease
C-myc: myelocytomatosis oncogene
FC: fold change
FISH: Fluorescence *in situ* hybridization
Fzd3: Frizzled family receptor 3
GFP: Green fluorescent protein
GSK-3β: Glycogen synthase kinase-3β
IBD: Inflammatory bowel disease
MMTV: Mouse mammary tumor virus
MSC: Mesenchymal stem cell
MSCT: Mesenchymal stem cell transplantation
Olfm4: Olfactomedin 4
PCR: Polymerase chain reaction
RT-PCR: Reverse transcription-polymerase chain reaction
Tcf4: T-cell factor 4
TNBS: 2,4,6-trinitrobenzene sulfonic acid
UC: Ulcerative colitis
Wnt3a: Wingless-type MMTV integration site family, member 3A
Wnt5a: Wingless-type MMTV integration site family, member 5A
Wnt11: Wingless-type MMTV integration site family, member 11
